# Prognostic impact of malignant diseases in idiopathic pulmonary fibrosis

**DOI:** 10.1038/s41598-020-75276-2

**Published:** 2020-10-26

**Authors:** Hong Yeul Lee, Jaeyoung Cho, Nakwon Kwak, Jinwoo Lee, Young Sik Park, Chang-Hoon Lee, Sang-Min Lee, Chul-Gyu Yoo, Young Whan Kim, Sun Mi Choi

**Affiliations:** Division of Pulmonary and Critical Care Medicine, Department of Internal Medicine, Seoul National University Hospital, 101 Daehak-ro, Jongno-gu, Seoul, 03080 Republic of Korea

**Keywords:** Diseases, Medical research, Oncology

## Abstract

No studies on idiopathic pulmonary fibrosis (IPF) have investigated the prognostic impact of extrapulmonary cancers in patients with IPF. We aimed to determine the prognostic impact of malignancies in patients with IPF. We retrospectively reviewed the medical records of patients diagnosed with IPF between 2001 and 2015. Patients were divided into three groups: IPF without cancer (n = 440), IPF with lung cancer (n = 69), and IPF with extrapulmonary cancer (n = 70). Of the 579 patients with IPF, 139 (24%) had cancer; the three most common types were lung (11.9%), gastric (2.4%), and colorectal (1.9%). Survival was significantly worse in patients with lung cancer than in those without cancer (hazard ratio [HR] = 1.83, 95% confidence interval [CI], 1.35–2.48) or those with extrapulmonary cancer (HR = 1.70, 95% CI, 1.14–2.54). The rate of hospitalisation for cancer-related complications was significantly higher in IPF patients with lung cancer than in those with extrapulmonary cancer. The annual rates of decline in percent predicted forced vital capacity and diffusion capacity for carbon monoxide did not differ among the groups. Physicians should pay attention to the development and progression of cancer and its prognostic impact in patients with IPF.

## Introduction

Idiopathic pulmonary fibrosis (IPF) represents the most common type of idiopathic interstitial pneumonia, and it occurs primarily in older adults^1^. Comorbidities are common in IPF patients, and they can negatively influence the quality of life and prognosis^2^. Pulmonary hypertension, chronic obstructive pulmonary disease, ischaemic heart disease, gastro-oesophageal reflux disease, and lung cancer are common comorbidities in IPF patients^2^. IPF and cancer share common risk factors, which include cigarette smoking, viral infections, environmental exposure, diabetes mellitus, and gastro-oesophageal reflux. They also have pathogenetic similarities^3,4^.

Several studies have reported that the incidence of lung cancer in patients with IPF was 11.2–36 cases per 1000 person-years^5–7^. A large cohort study of patients from the United Kingdom found a marked increase in the incidence of lung cancer in patients with IPF when compared with the general population (rate ratio 4.99; 95% confidence interval [CI], 3.03–8.22), but there was no increase in the incidence of other cancers. Moreover, the association between IPF and lung cancer persisted after adjustment for smoking (rate ratio 4.96; 95% CI, 3.00–8.18)^5^. A retrospective analysis reported that the survival rate was significantly worse in IPF patients with lung cancer than in those without lung cancer (median survival 38.7 months versus 63.9 months)^8^.

Most studies have focused on the prognostic impact of lung cancer in patients with IPF. However, there are limited data on extrapulmonary cancer in patients with IPF. Therefore, our study aimed to investigate the prognostic impact of extrapulmonary cancers in patients with IPF.

## Methods

### Study design and subjects

This retrospective cohort study involving patients with IPF was performed at Seoul National University Hospital, a tertiary teaching hospital in Seoul, South Korea. We reviewed all medical records and chest computed tomography images of patients diagnosed with IPF according to European Respiratory Society/American Thoracic Society criteria^4,9^. Patients aged > 18 years who were newly diagnosed with IPF between January 1, 2001, and December 31, 2015, and followed-up until December 31, 2017, were included. We excluded patients with certain causes of interstitial lung disease, including a history of domestic or occupational environmental exposure, drug toxicity, and connective tissue disease. Patients were divided into three groups: IPF without cancer (n = 440), IPF with lung cancer (n = 69), and IPF with extrapulmonary cancer (n = 70). Patients who were not diagnosed with a malignancy within 5 years before the IPF diagnosis until December 31, 2017, were included in the IPF without cancer group. Patients who were diagnosed with lung cancer or other malignancies within 5 years before the IPF diagnosis until December 31, 2017, were included in the IPF with lung cancer group or IPF with extrapulmonary cancer group, respectively. Cancer survivors usually have regular follow-ups scheduled at least every 5 years after the initial cancer diagnosis. Therefore, we chose this interval in our analysis. To ensure individual privacy, all the data analysed were anonymised and de-identified. The Institutional Review Board of the Seoul National University Hospital waived the requirement for written informed consent and approved this study. (Approval number IRB-H-1803-032-927). It was confirmed that all procedures adhered to the relevant guidelines and regulations. The present study conformed to the STROBE Statement.

### Data collection

All medical records were reviewed for IPF diagnosis, sex, comorbidities, smoking history and pack-years, body mass index (BMI), pulmonary function tests including absolute and percent predicted forced vital capacity (FVC), forced expiratory volume in 1 s (FEV_1_), FEV_1_/FVC ratio, and diffusing capacity for carbon monoxide (D_LCO_), chest computed tomography, cytological and pathological data, date of IPF diagnosis, date of malignancy diagnosis, number of emergency room (ER) visits and admissions to intensive care unit (ICU), and follow-up duration. The date of death was verified using several medical records and the death registry from the Ministry of the Interior and Safety, South Korea. All the cancers were diagnosed based on the clinical, radiological, and cytological or histopathological examinations. Based on cigarette smoking history, the study population was divided into smokers and non-smokers. Smokers were defined as current and ex-smokers who had smoked more than 100 cigarettes in their lifetime.

### Statistical analysis

Continuous baseline characteristics were summarised using the mean and standard deviation or median and interquartile range, whereas categorical characteristics were summarised using frequencies and percentages. Normally distributed quantitative data were compared using the analysis of variance. Quantitative data that were not normally distributed were compared using the Kruskal–Wallis test. Qualitative data were compared using the chi-squared test. The survival curves were estimated using the Kaplan–Meier method and were compared using the log-rank test. The univariate and multivariate Cox regression analyses were used to assess the prognostic impact of malignancies in patients with IPF. The hazard ratios (HRs) and 95% confidence intervals were estimated. All the analyses were two-tailed, and *p* values of < 0.05 were considered statistically significant. SPSS software version 25.0 for Windows (SPSS Inc., Chicago, IL, USA) was used for all statistical analyses.

## Results

### Clinical characteristics and prevalence of various cancers in IPF patients

A total of 579 patients newly diagnosed with IPF were included. Of them, 94 patients (16.2%) had undergone open lung biopsy to confirm the diagnosis of IPF. Among the patients with IPF, 139 (24%) patients were diagnosed with cancer; the five most common cancers were lung cancer (11.9%), gastric cancer (2.4%), colorectal cancer (1.9%), hepatocellular carcinoma (1.9%), and prostate cancer (1.0%). Table 1 shows the prevalence of the various malignancies. The patients were divided into three groups based on the diagnosis of malignancy: IPF without cancer (n = 440, 76%), IPF with lung cancer (n = 69, 11.9%), and IPF with extrapulmonary cancer (n = 70, 12.1%). The baseline clinical characteristics of IPF patients are shown in Table 2. For all patients with IPF, the median age at the time of IPF diagnosis was 68 years; 72.4% were men, mean BMI was 23.9, 63.9% were smokers, 33.9% had a history of hypertension, 26.6% had a history of diabetes mellitus, 16.6% had a history of tuberculosis, and the mean gender, age, and physiology score was 3.1 ± 1.4 points. Cancer comorbidities in IPF patients were more common in men and smokers. Patients with lung cancer had higher percent predicted FVC and predicted D_LCO_ as well as a lower FEV_1_/FVC ratio than those without cancer. Among the IPF patients with lung cancer, 2 patients (2.9%) were diagnosed with cancer before the IPF diagnosis, whereas 67 patients (97.1%) were diagnosed with cancer concomitantly (34.8%) or after (62.3%) the IPF diagnosis. The initial stages of lung cancer were as follows: 21 patients (30.4%) in stage I; 10 patients (14.5%) in stage II; 19 patients (27.5%) in stage III; and 19 patients (27.5%) in stage IV. The histologic types of lung cancer were squamous carcinoma in 24 patients (34.8%), adenocarcinoma in 23 patients (33.8%), unclassified non-small-cell lung cancer (NSCLC) in 12 patients (17.4%), small-cell lung cancer (SCLC) in 7 patients (10.1%), and large-cell lung cancer in 3 patients (4.3%). Among the IPF patients with extrapulmonary cancer, 20 patients (28.6%) were diagnosed with cancer before the IPF diagnosis, whereas 50 patients (71.4%) were diagnosed with cancer concomitantly (25.7%) or after (45.7%) the IPF diagnosis. Among the IPF patients with lung cancer stage I, 12 (57.1%), 8 (38.1%), and 1 (4.8%) were treated with surgery, radiotherapy, and chemotherapy, respectively. Further details on the first-line treatments stratified by lung-cancer stage are provided in Supplementary Table S1.

**Table 1:** Prevalence of malignancies in patients with idiopathic pulmonary fibrosis.

Malignancy	IPF cohort (n = 579)	
	Event	%
IPF without cancer	440	76.0
Lung cancer	69	11.9
Gastric cancer	14	2.4
Colorectal cancer	11	1.9
Hepatocellular carcinoma	11	1.9
Prostate cancer	6	1.0
Lymphoma, leukaemia	6	1.0
Pancreatobiliary cancer	4	0.7
Neuroendocrine tumour	3	0.5
Breast cancer	2	0.3
Multiple myeloma	2	0.3
Bladder cancer	2	0.3
Malignancy of undefined primary origin	2	0.3
Renal cell carcinoma	2	0.3
Cervical cancer	1	0.2
Thyroid cancer	1	0.2
Head and neck cancer	1	0.2
Skin cancer	1	0.2
Angiosarcoma	1	0.2

*IPF* idiopathic pulmonary fibrosis.

**Table 2:** Baseline characteristics of the study population.

Variables	IPF without cancer (n = 440)	IPF with lung cancer (n = 69)	IPF with extrapulmonary cancer (n = 70)	*p* value
Age at IPF diagnosis, years	68.0 (61.0–73.0)	69.0 (62.0–72.0)	68.0 (60.0–73.0)	0.978
Male	298 (67.7%)	64 (92.8%)	57 (81.4%)	< 0.001
Body mass index, kg·m^−2^	24.0 ± 3.1	24.0 ± 2.8	23.2 ± 2.9	0.080
*Smoking history*				
Smokers	246 (58.0%)	59 (86.8%)	53 (77.9%)	< 0.001
Smoking amount, pack-year	10.0 (0.0–30.0)	40.0 (23.5–50.0)	20.0 (10.0–40.0)	< 0.001
*Comorbidities*				
History of HT	148 (33.6%)	26 (37.7%)	22 (31.4%)	0.725
History of DM	121 (27.5%)	13 (18.8%)	20 (28.6%)	0.294
History of TB	74 (16.8%)	10 (14.5%)	12 (17.1%)	0.882
*Lung function*				
FVC, L	2.40 (1.92–3.01)	2.94 (2.53–3.44)	2.78 (2.41–3.31)	< 0.001
FVC, % pred	74.0 (61.0–87.0)	78.0 (72.0–93.0)	82.0 (69.0–96.0)	0.002
FEV_1_, L	1.98 (1.61–2.42)	2.35 (2.07–2.65)	2.24 (2.00–2.61)	< 0.001
FEV_1_, % pred	88.0 (74.0–103.0)	88.0 (82.0–103.0)	94.0 (76.0–114.0)	0.079
FEV_1_/FVC, %	83.0 (77.5–87.0)	79.0 (74.0–84.0)	82.0 (75.0–86.0)	< 0.001
D_LCO_, mL/mm Hg/min	10.3 (7.8–12.7)	12.5 (10.3–14.7)	11.5 (7.9–14.3)	< 0.001
D_LCO_, % pred	59.5 (48.0–75.0)	68.0 (56.0–84.0)	66.0 (50.0–86.0)	0.005
GAP score	3.1 ± 1.4	3.0 ± 1.2	3.0 ± 1.2	0.302

Data are presented as n (%), mean ± standard deviation, or median (interquartile range).

*D*_*LCO*_ diffusing capacity of the lung for carbon monoxide, *DM* diabetes mellitus, *FEV*_*1*_ forced expiratory volume in 1 s, *FVC* forced vital capacity, *GAP score* gender, age, and physiology score, *HT* hypertension; *IPF* idiopathic pulmonary fibrosis, *TB* pulmonary tuberculosis, *% pred* percent predicted.

### Prognostic impact of malignant diseases in IPF patients

**Figure 1:**
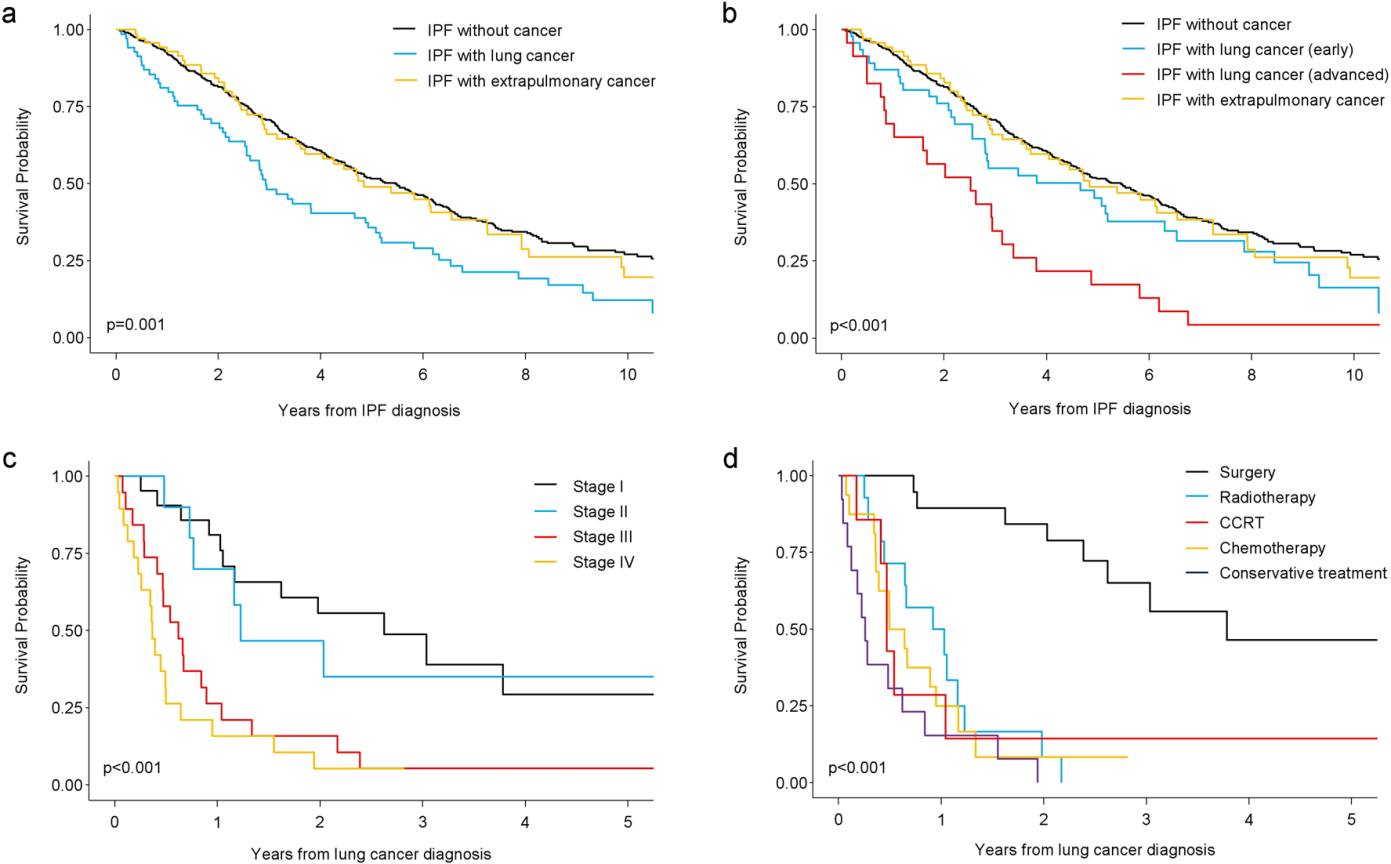
Kaplan–Meier curves for survival in patients with idiopathic pulmonary fibrosis (IPF) stratified by cancer status. (**a**) The IPF patients with lung cancer group are further classified into the early and advanced groups. (**b**) Subgroup analysis for survival in IPF patients with lung cancer according to the cancer stage at diagnosis (**c**) and first-line treatment after lung cancer diagnosis (**d**). *IPF* idiopathic pulmonary fibrosis.

Among the 579 patients with IPF, 382 (66.0%) died during the study period and the median survival time was 4.89 years (95% CI, 4.29–5.49). Among the IPF patients without cancer, 279 (63.4%) patients died and the median survival time was 5.45 years (95% CI, 4.73–6.18). Among the IPF patients with lung cancer, 57 (82.6%) patients died, and the median survival time was 2.93 years (95% CI, 2.30–3.56). Among the IPF patients with extrapulmonary cancer, 46 (65.7%) patients died, and the median survival time was 4.84 years (95% CI, 3.13–6.54). The 1-year, 5-year, and 10-year survival rates were 92.0%, 51.7%, and 27.1%, respectively, in the IPF group without cancer, 81.2%, 35.7%, and 12.2%, respectively, in the IPF group with lung cancer, and 92.9%, 49.0%, and 19.6%, respectively, in the IPF group with extrapulmonary cancer. Figure 1a shows the Kaplan–Meier estimates of the overall survival. There was a significant difference between the survival rates of the groups (*p* = 0.001). The log-rank test showed a significantly worse survival in the IPF group with lung cancer than in the group without cancer (*p* < 0.001) or with extrapulmonary cancer (*p* = 0.021). No significant difference was observed between the survival rates of IPF patients without cancer and those with extrapulmonary cancer (*p* = 0.726). The patients were divided into two groups based on the stage of cancer: early (stage I to IIIa NSCLC; limited-stage SCLC) and advanced (stage IIIb to IV NSCLC; extensive-stage SCLC). Further analysis revealed that patients with advanced-stage lung cancer had a significantly worse survival rate than those in the other groups (*p* < 0.001) (Fig. 1b). IPF patients with early-stage lung cancer did not have a worse survival rate than those without cancer (*p* = 0.075) or those with extrapulmonary cancer (*p* = 0.246). Among the IPF patients with lung cancer, the survival rate was worse in those with more advanced stages at diagnosis (Fig. 1c; *p* < 0.001), and the surgery group showed the most favourable survival (Fig. 1d; *p* < 0.001).

The univariate Cox regression analysis showed that lung cancer comorbidities, male sex, older age at the time of IPF diagnosis, lower BMI, percent predicted FVC lower than 80%, and percent predicted D_LCO_ lower than 80% were associated with worse survival in patients with IPF. In the multivariate analysis, which included variables with a *p* value of 0.20 or less in the univariate analysis, all of these factors remained significantly associated with worse survival in patients with IPF (Table 3). In the multivariate analysis, IPF patients with lung cancer had significantly worse survival than those without cancer (HR = 1.83; 95% CI, 1.35–2.48; *p* < 0.001) or those with extrapulmonary cancer (HR = 1.70; 95% CI, 1.14–2.54; *p* = 0.009). The survival rates of IPF patients without cancer and those with extrapulmonary cancer were not significantly different (HR = 1.08; 95% CI, 0.78–1.48; *p* = 0.652). Among IPF patients with lung cancer, Cox regression analysis adjusted for age, sex, BMI, percent predicted FVC, and percent predicted D_LCO_, revealed that the survival rate was significantly worse in those with more advanced stages at diagnosis, and those who received first-line treatment with surgery and concurrent chemoradiation therapy showed a more favourable survival rate than those who received conservative treatment (Table 4).

**Table 3:** Univariate and multivariate Cox regression analysis of the prognostic factors associated with survival in patients with idiopathic pulmonary fibrosis.

Variables	No. of patients	Univariate analysis		Multivariate analysis	
		HR (95% CI)	*p* value	HR (95% CI)	*p* value
*Malignancy*					
IPF without cancer	440	Ref		Ref	
IPF with lung cancer	69	1.69 (1.27–2.25)	< 0.001	1.83 (1.35–2.48)	< 0.001
IPF with extrapulmonary cancer	70	1.06 (0.77–1.44)	0.726	1.08 (0.78–1.48)	0.652
*Sex*					
Female	160	Ref		Ref	
Male	419	1.75 (1.37–2.24)	< 0.001	1.65 (1.26–2.15)	< 0.001
*Age at IPF diagnosis, years*					
< 65	217	Ref		Ref	
≥ 65	362	2.41 (1.93–3.02)	< 0.001	2.61 (2.06–3.31)	< 0.001
*Body mass index, kg·m*^*−2*^					
< 18.5	26	Ref		Ref	
≥ 18.5– < 25	349	0.30 (0.20–0.47)	< 0.001	0.39 (0.25–0.60)	< 0.001
≥ 25	204	0.23 (0.15–0.35)	< 0.001	0.30 (0.19–0.47)	< 0.001
*Smoking, pack-years*					
< 20	255	Ref			
≥ 20	263	1.14 (0.92–1.42)	0.226		
*Hypertension*					
None	383	Ref			
Present	196	0.96 (0.78–1.19)	0.707		
*Diabetes mellitus*					
None	425	Ref		Ref	
Present	154	1.22 (0.97–1.52)	0.084	1.06 (0.84–1.34)	0.609
*FVC, % pred*					
≥ 80	245	Ref		Ref	
< 80	333	1.64 (1.33–2.02)	< 0.001	1.51 (1.16–1.96)	0.002
*FEV*_*1*_*, % pred*					
≥ 80	387	Ref		Ref	
< 80	191	1.20 (0.97–1.48)	0.089	1.06 (0.82–1.38)	0.656
*FEV*_*1*_*/FVC, %*					
≥ 70	541	Ref			
< 70	37	0.80 (0.52–1.23)	0.313		
*D*_*LCO*_*, % pred*					
≥ 80	126	Ref		Ref	
< 80	439	1.36 (1.05–1.76)	0.021	1.68 (1.27–2.22)	0.001

*D*_*LCO*_ diffusing capacity of the lung for carbon monoxide, *FEV*_*1*_ forced expiratory volume in 1 s, *FVC* forced vital capacity, *HR* hazard ratio, *IPF* idiopathic pulmonary fibrosis, *% pred* percent predicted. All variables with a *p* value of 0.20 or less in the univariate analysis were included in the multivariate analysis.

**Table 4:** Cox regression analysis of the prognostic factors associated with survival in lung cancer patients with idiopathic pulmonary fibrosis.

Variables	No. of patients	Adjusted HR* (95% CI)	*p* value
*Lung cancer stage at diagnosis*			
Stage I	21	Ref	
Stage II	10	1.23 (0.49–3.10)	0.657
Stage III	19	3.79 (1.73–8.27)	0.001
Stage IV	19	5.17 (2.31–11.5)	< 0.001
*First-line treatment of cancer*			
Conservative treatment	13	Ref	
Surgery	19	0.09 (0.03–0.23)	< 0.001
Radiotherapy	14	0.56 (0.25–1.24)	0.149
CCRT	7	0.30 (0.10–0.91)	0.034
Chemotherapy	16	0.69 (0.30–1.59)	0.385

*CCRT* concurrent chemoradiation therapy, *HR* hazard ratio. *Adjusted for sex, age, body mass index, forced vital capacity (percent predicted), and diffusing capacity of the lung for carbon monoxide (percent predicted).

**Figure 2:**
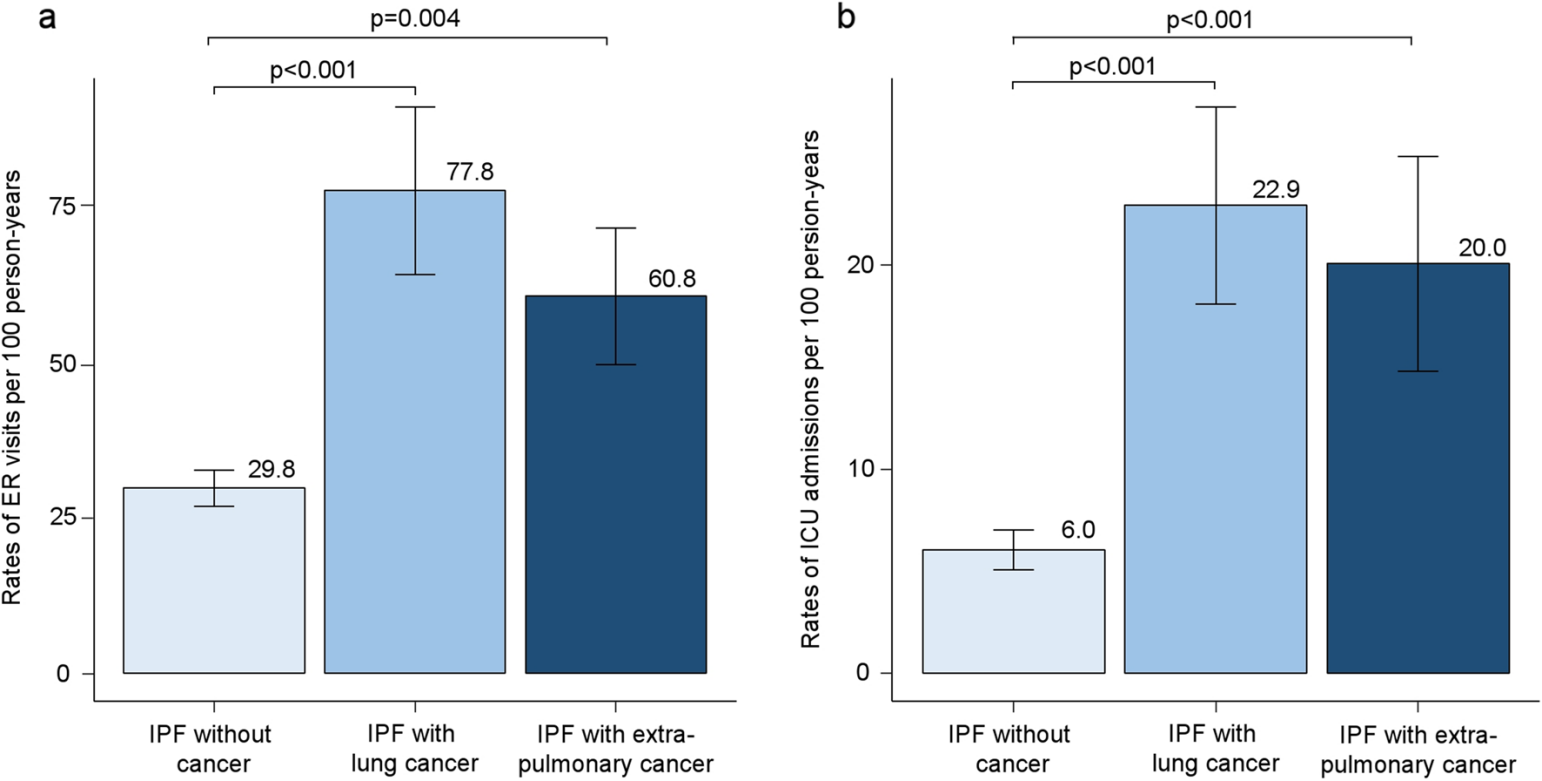
Rates of emergency room visit and admission to the intensive care unit per 100 person-years. (**a**) The differences in the rates of emergency room (ER) visits per 100 person-years among the groups. I bars indicate the standard error. (**b**) The differences in the rates of admission to the intensive care unit (ICU) per 100 person-years among the groups. I bars indicate the standard error. *IPF* idiopathic pulmonary fibrosis.

The overall rates of respiratory and non-respiratory hospitalisation among IPF patients per 100 person-years were 30.2 (95% CI, 25.4–35.0) and 21.6 (95% CI, 17.5–25.7), respectively. The rates of all-cause and non-respiratory hospitalisation were significantly higher among IPF patients with cancer than in those without cancer (*p* < 0.001 and *p* < 0.001, respectively) (Supplementary Table S2). The rate of respiratory hospitalisation was highest among IPF patients with lung cancer. The rate of hospitalisation for cancer-related complications was significantly higher in IPF patients with lung cancer than in those with extrapulmonary cancer (*p* = 0.011). The overall rates of ER visits and ICU admissions per 100 person-years were 39.3 (95% CI, 33.2–45.4) and 9.7 (95% CI, 7.4–12.0), respectively, in IPF patients. The rates of ER visits and ICU admissions were significantly higher among IPF patients with cancer than those without cancer (*p* < 0.001 and *p* < 0.001, respectively) (Fig. 2). In IPF patients without cancer, the rates of ER visits and ICU admissions per 100 person-years were 29.8 (95% CI, 24.1–35.5) and 6.0 (95% CI, 4.1–7.9), respectively. In patients with lung cancer, the rates of ER visits and ICU admissions per 100 person-years were 77.8 (95% CI, 50.7–104.8) and 22.9 (95% CI, 13.2–32.5), respectively. The rates of ER visits and ICU admissions per 100 person-years in patients with extrapulmonary cancer were 60.8 (95% CI, 38.8–82.7) and 20.0 (95% CI, 9.5–30.5), respectively. However, the rates of ER visits and ICU admissions of IPF patients with lung cancer and those with extrapulmonary cancer were not significantly different (*p* = 0.389 and *p* = 0.826, respectively). Of the IPF patients, 54.0%, 27.3%, and 11.0% were admitted to the ICU for respiratory causes, postoperative management, and cardiovascular causes, respectively. Of 111 IPF patients who died in the hospital, 64 patients (57.7%) had end-of-life discussions with their physicians and refused ICU admission. There was no significant difference between the proportions of patients who refused ICU admission among the groups (*p* = 0.189). The overall ICU mortality rate was 24.2% in patients with IPF; there was no significant difference in the ICU mortality rates of the groups (*p* = 0.256).

### Lung function

**Figure 3:**
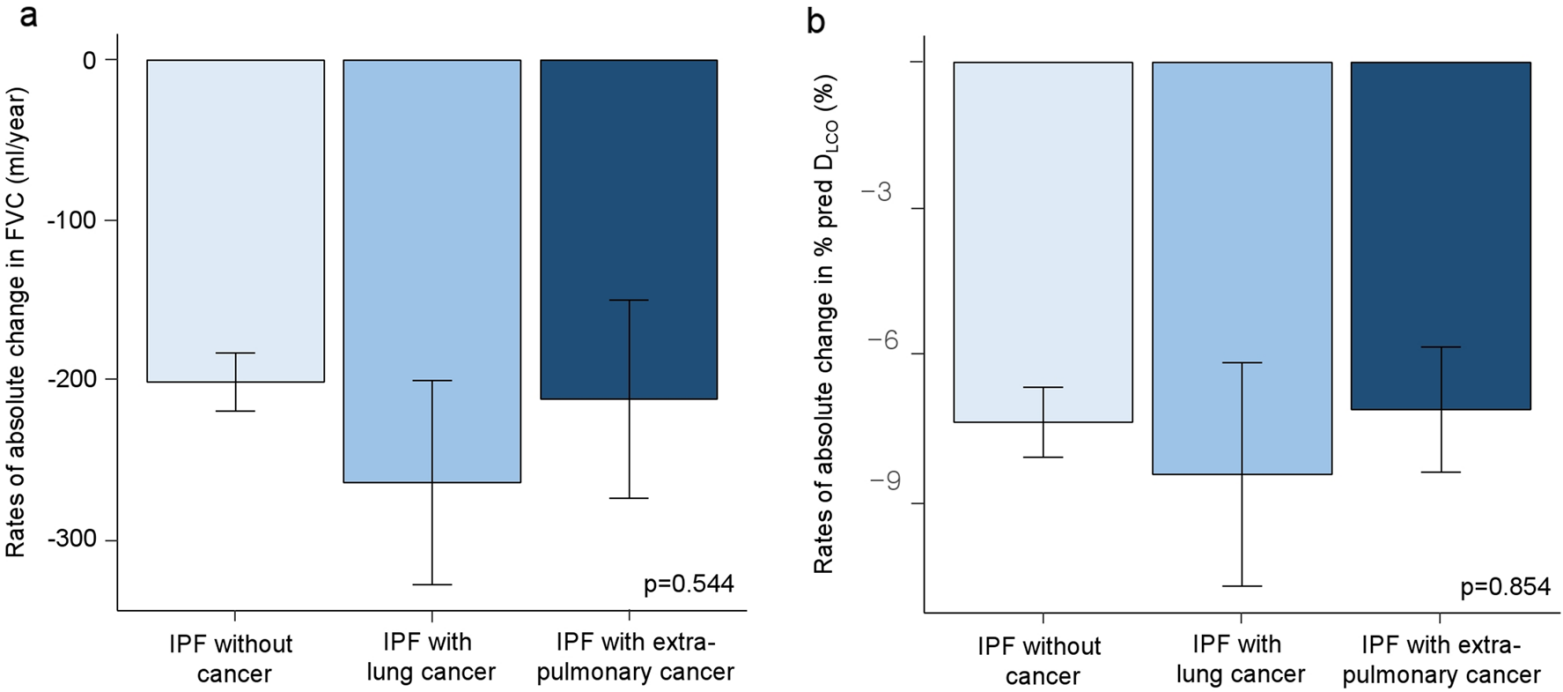
Annual rates of absolute change from the baseline in FVC and D_LCO_. (**a**) The differences in the annual rates of absolute change in FVC among the groups. I bars indicate the standard error. (**b**) The differences in the annual rates of absolute change in percent predicted D_LCO_ among the groups. I bars indicate the standard error. *IPF* idiopathic pulmonary fibrosis, *FVC* forced vital capacity, *D*_*LCO*_ diffusing capacity of the lung for carbon monoxide, *% pred* percent predicted.

Figure 3 shows the annual rates of change in FVC and D_LCO_. In IPF patients without cancer, the annual rates of absolute change in percent predicted FVC, FVC (ml/year), and percent predicted D_LCO_ from the baseline were − 5.2 ± 11.0% per year, − 201.9 ± 36.4 ml per year, and − 7.4 ± 14.1% per year, respectively. In patients with lung cancer, the rates were − 6.7 ± 12.6% per year, − 264.7 ± 47.0 ml per year, and − 8.5 ± 16.7% per year, respectively. In patients with extrapulmonary cancer, the rates were − 5.5 ± 12.2% per year, − 212.7 ± 48.6 ml per year, and − 7.1 ± 9.8% per year, respectively. The annual rates of absolute change in percent predicted FVC, FVC (ml/year), and percent predicted D_LCO_ from the baseline did not differ significantly among the groups (*p* = 0.672, *p* = 0.544, and *p* = 0.854, respectively).

## Discussion

The present retrospective longitudinal cohort study revealed that the prevalence of cancer in IPF patients was 24% and that of lung cancer was 11.9%. Lung cancer was the most common cancer type among IPF patients. Lung cancer comorbidity was an independent prognostic factor for poor survival in patients with IPF, regardless of age, sex, BMI, percent predicted FVC, and percent predicted D_LCO_. However, extrapulmonary cancer comorbidity was not associated with poor survival in patients with IPF. The overall rates of ER visits, all-cause hospitalisation, and ICU admission were significantly higher among IPF patients with cancer than those without cancer. However, the rate of hospitalisation for cancer-related complications was significantly higher in IPF patients with lung cancer than in those with extrapulmonary cancer. The annual rates of absolute change in FVC and D_LCO_ from the baseline did not differ significantly among the groups.

Previous studies have shown that lung cancer in IPF patients was more common in men and smokers. IPF patients with lung cancer had higher FVC and lower FEV_1_/FVC than those without cancer^6,8,10,11^. These results are consistent with those of this study. Cigarette smoking has been reported as the major cause of emphysema and various cancers^12,13^, and patients with combined pulmonary fibrosis and emphysema had significantly higher FVC and lower FEV_1_/FVC than those with pulmonary fibrosis alone^11,14^. These are similar to our findings on IPF patients with cancer.

Our analysis showed that the prevalence of cancer among IPF patients was 24%, and lung cancer was the most common cancer (11.9%), followed by gastric cancer (2.4%), colorectal cancer (1.9%), hepatocellular carcinoma (1.9%), and prostate cancer (1.0%). There are some variations in the published prevalence of lung cancer in patients with IPF, ranging from 4.8% to 48%. These values are significantly higher than those in patients without IPF (2.0% to 6.4%)^15^. A recent meta-analysis, which included 131,947 patients with IPF from 35 studies, reported that the overall lung cancer prevalence was 13.5% (95% CI, 10.4–17.4)^16^. However, data on extrapulmonary cancers in patients with IPF are limited. An observational retrospective long-term analysis of IPF patients who were screened for comorbidities reported that out of 272 patients with IPF, 60 (22.1%) had cancer, 42 (15.4%) had lung cancer, and 18 (6.6%) had extrapulmonary cancers^17^. These results are similar to ours. The mechanisms of the development of cancer in IPF patients remain unclear. Some mechanisms have been proposed for the association between IPF and cancer. IPF and cancer share common risk factors, especially cigarette smoking^3^. Cigarette smoking is a well-known major risk factor for the development of various cancers^12^. The International Agency for Research on Cancer report shows that there is sufficient evidence on the carcinogenicity of cigarette smoking in humans^18^. Moreover, IPF and cancer have pathogenetic similarities, such as epigenetic and genetic abnormalities, abnormal activation of specific signal transduction pathways, and altered cell-to-cell communications^3^. However, due to the small sample size and the design of this study, the association between IPF and cancer development could not be fully analysed and explained. Further well-designed, population-based studies are needed to compare the risks of various cancers in patients with and without IPF.

Previous studies have reported several predictors of poor survival in IPF at baseline, which include older age, male sex, worse dyspnoea, lower FVC, lower D_LCO_, lower BMI, need for supplemental oxygen, greater extent of fibrosis on chest computed tomography, and shorter distance on the six-minute walk test^19–23^. Similarly, the multivariate Cox proportional hazard model in the present study showed that older age, male sex, lower BMI, percent predicted FVC lower than 80%, and percent predicted D_LCO_ lower than 80% at baseline were associated with poor survival in patients with IPF. In addition, our results showed that IPF patients with lung cancer had a significantly poor survival than those without cancer or those with extrapulmonary cancer, even after multivariable adjustment for other known predictors of poor survival. Several studies have reported that the presence of lung cancer among IPF patients was an independent predictor of poor survival^8,10,17^. A previous study from Italy reported that survival in IPF patients was worse in those with than those without lung cancer (HR = 5.0; 95% CI, 2.91–8.57; *p* < 0.001). Causes of death in this study were respiratory failure (43%), lung cancer treatment-related complications (17%), and progression of lung cancer (13%)^8^. In a previous retrospective long-term analysis of IPF patients, lung cancer comorbidity was associated with poor survival (HR = 2.92; 95% CI, 1.62–5.28; *p* < 0.001), while extrapulmonary cancer comorbidity had no impact on survival in patients with IPF (HR = 1.15; 95% CI, 0.57–2.31; *p* = 0.702); this was similar to our results^17^. In this study, IPF patients with early-stage lung cancer and those without cancer or with extrapulmonary cancer had comparable survival rates. Additionally, among IPF patients with lung cancer, the survival rate was worse in those with more advanced stages at diagnosis, and those who received first-line treatment with surgery showed the most favourable survival. Currently, there are no recommendations on how to manage IPF patients with lung cancer. A recent study showed that surgery was effective for lung cancer in patients with IPF, which was consistent with our results^24^. Accordingly, active treatment, such as surgical treatment, may be effective for IPF patients with early-stage lung cancer. The findings from this study add to the evidence that lung cancer comorbidity is an independent prognostic factor for poor survival in patients with IPF, while extrapulmonary cancer comorbidity is not associated with poor survival in patients with IPF.

The data on emergency department visits, ICU admissions, and annual change in lung function in IPF patients with cancer are limited in the literature. In this study, overall rates of ER visits and ICU admissions were significantly higher among IPF patients with cancer than those without cancer, while annual rates of absolute change in FVC and D_LCO_ from the baseline did not differ significantly among the groups. A previous systematic review reported that the rates of emergency department visits of cancer patients exceeded those of the general population, which was consistent with our results^25^. A previous study reported that hospitalisation rates and long-term mortality rates of IPF patients admitted to the ICU were high^26^. Although our results showed that the rates of ER visits, all-cause hospitalisation, and ICU admission were significantly higher among IPF patients with cancer than those without cancer, patients with lung cancer showed poorer survival than the other groups. Previous studies found that the difference between the survival rates of IPF patients with lung cancer and those without cancer was not due to the worsening of pulmonary fibrosis; it was mainly due to lung cancer progression and cancer treatment-related complications^8,27^. Interestingly, our results showed that the rate of hospitalisation for cancer-related complications was significantly higher in IPF patients with lung cancer than those with extrapulmonary cancer. Additionally, the rate of respiratory hospitalisation was highest among IPF patients with lung cancer. These findings suggest that the higher incidence of cancer-related complications and the accompanying rate of respiratory hospitalisation may account for survival differences among the groups.

Our study has several limitations. Due to the retrospective nature of the study, inadequate or missing data could have affected the outcome. However, we used the death registry from the Korean Ministry of the Interior and Safety. Hence, there were no missing data on the date of death. Our data were obtained from a single hospital, and the sample size was relatively small for evaluating the exact prevalence of each type of cancer in patients with IPF. In addition to the low prevalence of IPF, cancer comorbidity among IPF patients is less common. Therefore, further population-based studies are needed to identify the exact prevalence of various cancers in patients with IPF and their prognostic impact. The cause of death could not be determined in some patients because the records for out-of-hospital deaths were unreliable.

In conclusion, this study revealed that the prevalence of cancer among IPF patients was 24%. The overall rates of ER visits, all-cause hospitalisation, and ICU admission were significantly higher among IPF patients with cancer than those without cancer. However, the rate of hospitalisation for cancer-related complications was significantly higher in IPF patients with lung cancer than in those with extrapulmonary cancer. IPF patients with lung cancer had a worse survival than those with extrapulmonary cancer. Interestingly, IPF patients with early-stage lung cancer or extrapulmonary cancer, as well as those without cancer, had comparable survival rates. From these results, physicians should pay attention to the development and progression of cancer and its prognostic impact in patients with IPF. Further population-based studies are needed to validate these results.

## Supplementary information

Supplementary Tables

## Data Availability

The datasets generated during the current study are available from the corresponding author on request.
