# Supplementary material for: Prognostic impact of malignant diseases in idiopathic pulmonary fibrosis
Source: Sci Rep. 2020 Oct 26;10:18260. doi: 10.1038/s41598-020-75276-2 (PMC7588444; doi:10.1038/s41598-020-75276-2)
Supplement: Supplementary file 1 — Supplementary Tables [file 41598_2020_75276_MOESM1_ESM.docx]

**Prognostic impact of malignant diseases in idiopathic pulmonary fibrosis**

**Short title:** Prognostic impact of malignant diseases in IPF

**Author list:**

Hong Yeul Lee^1^, Jaeyoung Cho^1^, Nakwon Kwak^1^, Jinwoo Lee^1^, Young Sik Park^1^, Chang-Hoon Lee^1^, Sang-Min Lee^1^, Chul-Gyu Yoo^1^, Young Whan Kim^1^, Sun Mi Choi^1*^

**Affiliations:**

^1^Division of Pulmonary and Critical Care Medicine, Department of Internal Medicine, Seoul National University Hospital, Seoul, Republic of Korea.

***Corresponding author:**

Sun Mi Choi, MD,

Address: Seoul National University Hospital, 101 Daehak-ro, Jongno-gu, Seoul 03080, Republic of Korea.

Tel: +82-2-2072-4915

Fax: +82-2-762-9662

E-mail: sunmich81@gmail.com

Supplementary Table S1. Comparison of first-line treatment modalities stratified by lung cancer stage in patients with idiopathic pulmonary fibrosis

| **Variables** | **Stage I (n = 21)** | **Stage II (n = 10)** | **Stage III (n = 19)** | **Stage IV (n = 19)** |
| --- | --- | --- | --- | --- |
| **Surgery** | 12 (57.1%) | 6 (60.0%) | 1 (5.3%) | 0 |
| **Radiotherapy** | 8 (38.1%) | 2 (20.0%) | 3 (15.8%) | 1 (5.3%) |
| **CCRT** | 0 | 0 | 7 (36.8%) | 0 |
| **Chemotherapy** | 1 (4.8%) | 1 (10.0%) | 5 (26.3%) | 9 (47.4%) |
| **Conservative treatment** | 0 | 1 (10.0%) | 3 (15.8%) | 9 (47.4%) |

CCRT: concurrent chemoradiation therapy

Supplementary Table S2. Rates of hospitalisation per 100 person-years in patients with idiopathic pulmonary fibrosis

| **Variables** | **IPF without cancer (95% CI)** | **IPF with lung cancer (95% CI)** | **IPF with extrapulmonary cancer (95% CI)** | **p-value** |
| --- | --- | --- | --- | --- |
| **All-cause** | 36.7 (30.8–42.6) | 106.3 (82.8–129.6) | 93.0 (65.2–120.9) | < 0.001 |
| **Respiratory** | 27.2 (21.9–32.4) | 48.7 (31.8–65.6) | 30.9 (16.5–45.5) | 0.018 |
| **Non-respiratory** | 9.5 (7.3–11.8) | 57.6 (41.6–73.7) | 62.1 (39.0–85.2) | < 0.001 |
| **Cancer-related complications** |  | 41.4 (26.2–56.6) | 18.0 (8.1–27.9) | 0.011^*^ |

IPF: idiopathic pulmonary fibrosis. *Comparison between IPF patients with lung cancer and those with extrapulmonary cancer.
